# Long-term prognostic value of staging surgery for high-intermediate-risk and high-risk endometrial cancer

**DOI:** 10.1007/s11701-026-03316-6

**Published:** 2026-04-27

**Authors:** Alise de Jong, Jasper Markus, Ronald P. Zweemer, Jacob P. Hoogendam, Judith M. Roesink, Inge O. Baas, Geertruida N. Jonges, Cornelis G. Gerestein

**Affiliations:** 1 Department of Gynecologic Oncology, University Medical Center Utrecht, Utrecht University, PO Box 85500, 3508 GA Utrecht, The Netherlands; 2 Department of Radiotherapy, University Medical Center Utrecht, Utrecht University, Heidelberglaan 100, 3584 CX Utrecht, The Netherlands; 3 Department of Medical Oncology, University Medical Center Utrecht, Utrecht University, 85500, 3508 GA Utrecht, The Netherlands; 4 Department of Pathology, University Medical Center Utrecht, Utrecht University, 85500, 3508 GA Utrecht, The Netherlands

**Keywords:** Endometrial neoplasms, Robotic surgical procedures, Treatment outcome, Disease-free survival, Recurrence

## Abstract

The prognostic relevance of surgical staging in patients with high-intermediate-risk and high-risk endometrial cancer remains uncertain. In this cohort study, we investigated the prognostic role of robot-assisted laparoscopic staging surgery among patients with clinically early-stage endometrial carcinoma at a high-intermediate-risk or high-risk for recurrence. Clinical data of women with clinically International Federation of Gynaecology and Obstetrics (FIGO) 2009 stage I–II, grade 3 endometrioid or non-endometrioid EC who were intended to undergo robot-assisted laparoscopic staging surgery were retrospectively collected from a single tertiary referral center. The procedure consisted of total hysterectomy with bilateral salpingo-oophorectomy, pelvic lymphadenectomy, and, when feasible, para-aortic lymphadenectomy, with additional omentectomy and peritoneal biopsies performed in a subset of cases. Survival outcomes were assessed using Kaplan–Meier analysis, with additional subgroup analyses by histological subtype. A total of 166 patients, of which 154 patients (92.8%) with FIGO 2009 stage I and 12 patients (7.2%) with FIGO 2009 stage II were included, comprising various histological subtypes, including 64 (38.6%) with endometrioid carcinoma, 52 (31.3%) with serous carcinoma, 11 (6.6%) with clear cell carcinoma, and 27 (16.3%) with carcinosarcoma. Thirty-two patients (19.3%) were reclassified as having FIGO stage disease III–IV based on final pathology. The 5-year disease-specific survival was 25.5% for upstaged patients compared with 73.1% for those who were not upstaged. Robot-assisted laparoscopic staging provides valuable prognostic information in clinically early-stage endometrial cancer with a high-intermediate-risk or high-risk of recurrence. These findings underscore the value of surgical staging in informing prognosis and guiding adjuvant treatment decisions.

**Supplementary Information:**

The online version contains supplementary material available at 10.1007/s11701-026-03316-6.

## Introduction

Endometrial cancer (EC) represents a major global health burden, ranking seventh among the most frequently diagnosed cancers in women worldwide and fourth among European women [1]. Approximately 420,300 new cases of EC were reported globally in 2022, of which nearly 125,000 occurred within Europe [2, 3]. Both the incidence and mortality rates of EC have continued to increase, largely attributable to the growing prevalence of established risk factors [4–6].

The World Health Organization (WHO) classifies EC into several main histologic tumour subtypes, including endometrioid, serous, and clear cell carcinoma (CCC), undifferentiated (UC) and dedifferentiated carcinomas, carcinosarcoma (CS), and mixed carcinoma [1, 5, 6]. The International Federation of Gynaecology and Obstetrics (FIGO) staging system further classifies EC by anatomical characteristics. In 2023, the FIGO classification system was updated to integrate molecular classification with tumour characteristics and histological subtype [7].

To further determine stage, staging surgery is recommended for patients with grade 3 endometrioid endometrial carcinoma (EEC) or non-endometrioid EC [1, 7–9]. In staging surgery, pelvic lymph node dissection (PLND) with or without paraaortic lymph node dissection (PALND) is performed in addition to hysterectomy and bilateral salpingo-oophorectomy. In serous carcinoma (SC) and CS omentectomy and peritoneal biopsies should be considered [10]. Surgical staging plays an important role in determining which patients require intensive adjuvant treatment. It provides insight into predictors of prognosis, such as lymph node status, lymphovascular space invasion (LVSI) and dept of myometrial invasion [11, 12]. This can guide adjuvant treatment strategies and identify patients in whom adjuvant treatment may be safely omitted. However, it remains unclear whether the prognostic information obtained through (robot-assisted laparoscopic) staging surgery has an impact on overall survival (OS), disease-specific survival (DSS) or disease-free survival (DFS) [13]. In this cohort study, we evaluated the prognostic role of robot-assisted laparoscopic staging (RALS) surgery among patients with clinically early-stage EC at a high-intermediate-risk or high-risk for recurrence.

## Methods

All women diagnosed with clinically FIGO 2009 stage I or II, grade 3 EEC or non-endometrioid EC who were intended to treat with RALS surgery between the 1st of January, 2012, until the 31st of December, 2023, at the University Medical Center Utrecht, were retrospectively included. Patients were treated in accordance with contemporaneous national and international guidelines. Follow-up information was documented in the medical files. Given the retrospective design and use of pseudonominysed data, formal informed consent was not required, as approved by the institutional review board.

The primary aim is to evaluate the prognostic role of RALS among patients with clinically early-stage EC, with presumed high-intermediate-risk or high-risk for recurrence. Main objectives are to determine in how many patients RALS resulted in upstaging to advanced FIGO stages (FIGO 2009 stage III or IV) and to determine OS, DSS, and DFS for patients in whom RALS resulted in upstaging to advanced FIGO stages III or IV and compare results to patients without upstaging.

Secondary objectives are to determine which were the localisations of metastases leading to upstaging. Other objectives include assessing differences in OS, DSS and DFS between patients who were upstaged and those who were not for each histological subtype.

Patients eligible for inclusion were 18 years old or older, with a diagnosis of primary endometrial carcinoma (grade 3 EEC, SC, CCC, CS, UC and dedifferentiated carcinomas or mixed carcinoma) confirmed by histopathological examination, pre-operative early FIGO 2009 stage (defined as stage I or II) who were treated primarily with RALS surgery. Patients receiving neoadjuvant treatment prior to surgery were excluded. Relevant clinical variables, including demographics, tumour characteristics, surgical treatment, and follow-up data, were obtained from electronic patient files.

Risk group classification was performed in accordance with the European Society of Gynaecological Oncology/European Society for Radiotherapy and Oncology/European Society of Pathology (ESGO/ESTRO/ESP) guidelines of 2020 [14]. During the study period, complete molecular classification was not routinely available. Patients with preoperative stage I grade 3 EEC and stage II EEC were classified as high-intermediate risk EC, whereas preoperative stage I or II EC with non-endometrioid histologies (SC, CCC, UC and dedifferentiated carcinomas, CS, or mixed carcinoma) with myometrial invasion were classified as high-risk EC. Standard preoperative imaging consisted of transvaginal ultrasonography and abdominal and thoracic computed tomography (CT). Preoperative magnetic resonance imaging was not routinely performed to assess for myometrial invasion.

The surgical procedure regarding patients with clinically early-stage EC at high-intermediate- or high-risk for recurrence, performed at the UMC Utrecht has been previously described [15]. Surgery was performed by a team of three gynaecologic oncologists using a robotic surgical system (da Vinci Surgical System Si until 2017, X or Xi from 2018–2023; Intuitive Surgical, Sunnyvale, CA). RALS surgery comprised of total hysterectomy with bilateral salpingo-oophorectomy, PLND, and, when feasible, PAOLND. PLND included systematic excision of lymphatic tissue along the common, external and internal iliac vessels and in the obturator fossa. PAOLND comprised excision of precaval and paracaval lymphatic tissue up to the level of the left renal vein. During this study period, the sentinel lymph node (SLN) procedure was not standard of care according to contemporaneous guidelines. In CSa, SCs or CCC histologies, omentectomy and peritoneal tissue sampling were performed in addition to standard staging. Surgical methods used, including use of the McCartney uterine manipulator manufactures by LiNA Medical (Denmark), remained unchanged throughout the study period. Operation time was calculated from incision until the skin was sutured. Adjuvant treatment strategies were determined in a multidisciplinary tumour board setting in accordance with contemporary guidelines and were risk-adapted based on postoperative FIGO 2009 stage, histological subtype, tumour grade, depth of myometrial invasion, LVSI, nodal involvement, and patient-related factors. Oncological follow-up was conducted according to national guidelines, with visits scheduled at three-monthly intervals during the first two years after surgery, followed by six-monthly intervals thereafter, up to five years post-treatment. Follow-up visits alternated between the gynaecologist and the radiation oncologist or medical oncologist, and no routine CT scans or tumour marker assessments were performed.

Continuous variables were described using means with standard deviation (SD) or medians with interquartile range (IQR) depending on data distribution. Categorial variables were reported as counts and percentages. Images were created with Biorender.com.

OS was defined as the interval, in days, from the date of the operation to the date of death from any cause or the date of last-follow-up. DSS was defined as the interval from the date of the operation to the date of death of disease or the date of last follow-up. Non–disease deaths are censored at death for DSS. DFS was defined as the interval from the operation date until formal confirmation of recurrence at a multidisciplinary tumour board meeting, or until the most recent follow-up. Time-to-event outcomes were evaluated using Kaplan–Meier survival analysis. Statistical significance was assessed using log-rank tests with administrative censoring at 12, 36, and 60 months. A *p*-value < 0.05 was defined as statistically significant. Standard error was determined using Greenwood’s formula. Cox proportional hazards regression was performed to assess whether upstaging remained independently associated with survival after adjustment for confounders. Multivariable models included age, LVSI, and myometrial invasion, selected based on clinical relevance and univariable significance, with the number of covariates restricted to avoid overfitting. Hazard ratios (HRs) with 95% confidence intervals (CIs) were reported. All analysis, figures and plots were generated in the R statistical computing environment (R version 4.5.1R Core Team, 2025).

## Results

Clinical characteristics of the 166 included patients with clinically early-stage EC at a high-intermediate- or high-risk for recurrence are described in Table 1. Follow-up duration was 25 months [IQR 13.0–44.8].

**Table 1:** Baseline characteristics of study population

Characteristic	Study population, *n* = 166
Age, years	68.50 (62.00, 73.00)
BMI, kg/m^2^	27.48 (23.74, 31.89)
Previous abdominal infection	31 (18.7%)
Previous abdominal surgery	85 (51.2%)
Smoking	
*Current*	18 (10.8%)
*Stopped over ten years ago*	8 (4.8%)
*Stopped over five years ago*	1 (0.6%)
*Stopped less than five years ago*	5 (3.0%)
*Stopped*	4 (2.4%)
*Never*	130 (78.3%)
Preoperative histology	
*Endometrioid carcinoma*	67 (40.4%)
*Serous carcinoma*	58 (34.9%)
*Clear cell carcinoma*	14 (8.4%)
*Carcinosarcoma*	18 (10.8%)
*Undifferentiated carcinoma*	5 (3.0%)
*Other*	4 (2.4%)
Preoperative FIGO 2009	
*IA*	141 (84.9%)
*IB*	13 (7.8%)
*II*	12 (7.2%)
PLND performed	160 (96.4%)
PALND performed	136 (81.9%)
Lymph nodes dissected, n	20 (14, 23)
Omentectomy performed	108 (65.1%)
Peritoneum biopsy performed	91 (54.8%)
Operation time, min	230 (195, 267)
*Unknown*	1
Blood loss, ml	100 (50, 150)
*Unknown*	15

Statistics presented: median (interquartile range); n (%)

BMI = body mass index, PLND = pelvic lymph node dissection, PALND = para‑aortic lymph node dissection, ml = milliliters, min = minutes, FIGO = International Federation of Gynecology and Obstetrics

160 patients (96.4%) underwent PLND. 136 patients (81.9%) underwent PALND. The median BMI was 33.96 (30.47–39.04) in the group without PALND, compared to 25.83 (29.38–23.31) in the group that underwent PALND (Table 1).

**Fig. 1:**
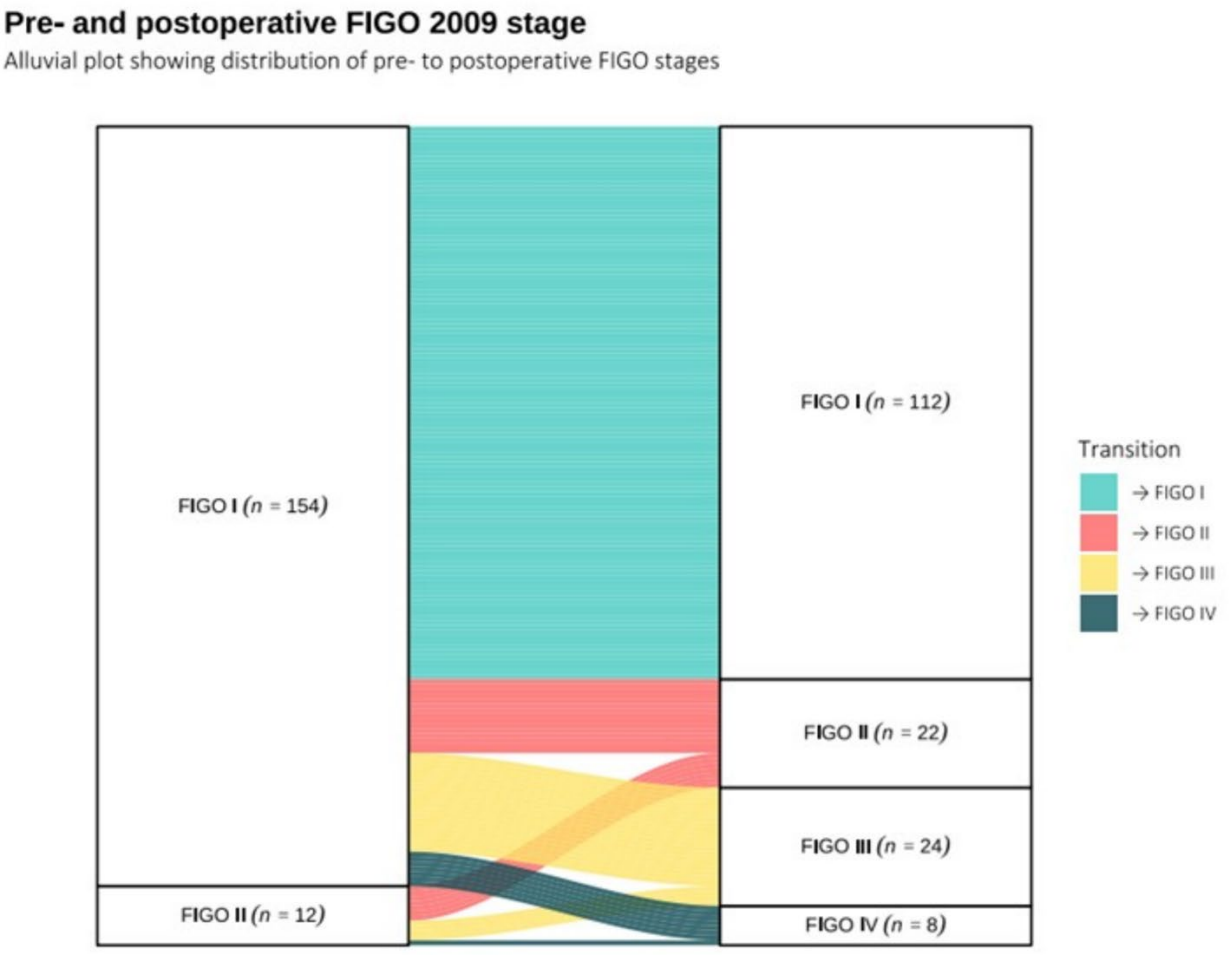
Pre- and postoperative FIGO 2009 stage

Following RALS, 32/166 included patients (19.3%) were reclassified as having advanced stage FIGO III-IV disease after surgical staging and were therefore considered upstaged (Fig. 1) In total, 21/32 patients (65.6%) had metastatic lymph nodes; only 5/21 patients (23.8%) had isolated para-aortic lymph node metastasis. 5/32 patients (15.6%) had omental metastasis and 5/32 patients (15.6%) had peritoneum metastasis. Patients were upstaged due to the following findings: 2/32 (6.3%) because of both omental and peritoneal metastases, 3/32 (9.4%) due to omental metastases only, and 3/32 (9.4%) due to peritoneal metastases only. Among these eight patients, 4/8 (50%) had parametrial involvement and 3 (37.5%) also had positive lymph nodes. An additional 18/32 patients (56.3%) were upstaged due to lymph node metastases, of whom 3/18 (16.7%) also showed parametrial involvement. Finally, 4/32 patients (12.5%) were upstaged due to parametrial involvement alone, 1/32 (3.1%) due to vaginal involvement, and 1/32 (3.1%) due to adnexal invasion (Table 2).

**Table 2:** Postoperative pathology characteristics of study population

Characteristic	Study population, *n* = 166
Upstaged to FIGO stage III–IV	32 (19.3%)
Risk classification^1^	
*Low*	3 (1.8%)
*Intermediate*	34 (20.9%)
*High‑intermediate*	32 (19.6%)
*High*	94 (57.7%)
*Unknown*	3
Postoperative histology	
*Endometrioid carcinoma*	64 (38.6%)
*Serous carcinoma*	52 (31.3%)
*Clear cell carcinoma*	11 (6.6%)
*Carcinosarcoma*	27 (16.3%)
*Undifferentiated carcinoma*	4 (2.4%)
*Other*	6 (3.6%)
*Tumor free*	2 (1.2%)
Lymph node metastasis	
*Yes*	21 (12.7%)
*Micrometastasis*	18 (10.7%)
*Macrometastasis*	3 (1.8%)
*No*	141 (84.9%)
*Not performed*	4 (2.4%)
Omental metastasis	
*Yes*	5 (3.0%)
*No*	103 (62.0%)
*Not performed*	58 (34.9%)
Peritoneal metastasis	
*Yes*	5 (3.0%)
*No*	86 (51.8%)
*Not performed*	75 (45.2%)
Adjuvant therapy	
*None*	25 (15.1%)
*Vaginal brachytherapy*	95 (57.2%)
*External beam radiotherapy*	30 (18.1%)
*Chemotherapy*	11 (6.6%)
*Other*	5 (3.0%)

Statistics presented: median (interquartile range); n (%)

ESGO = European Society of Gynaecological Oncology, ESTRO = European Society for Radiotherapy and Oncology, ESP = European Society of Pathology, FIGO = International Federation of Gynecology and Obstetrics

^1^ESGO/ESTRO/ESP guidelines 2020

The majority of patients who remained classified as early-stage FIGO stage I-II disease underwent vaginal brachytherapy (VBT) (*n* = 94/134, 70.1%) postoperatively. The majority of patients who were reclassified as having advanced stage FIGO III-IV disease received external beam radiotherapy (EBRT) (*n* = 13/32, 40.6%). 9/32 patients (28.1%) underwent adjuvant chemotherapy. Adjuvant therapy was not administered in 6/32 patients(18.8%) due to rapid disease progression in two patients, refusal of adjuvant therapy in two patients who subsequently received hormonal therapy or chemotherapy at disease relapse, another patient being judged unfit for adjuvant treatment, and follow-up information missing for one patient (Supplementary 1).

OS rates for the 134 patients who remained classified as early-stage FIGO stages I-II were 93.4%, 82.5% and 67.5%, compared to 68%, 34.9% and 25.5% in the upstaged group at 1, 3 and 5-year follow-up, respectively (*p*-value < 0.01). DSS rates in patients who remained classified as early-stage FIGO stages I-II were 93.4%, 83.4% and 73.1% at 1, 3 and 5-year follow-up, respectively (*p*-value < 0.01). DSS rates did not differ from overall survival among patients who were reclassified as having advanced FIGO stages III or IV disease (Table 3).

**Table 3:** Survival rates

Survival type	Timepoint	Study population (*n* = 166)			Postoperative high-(intermediate-) risk group (*n* = 106)		
		Not upstaged (*n* at risk)	Upstaged (*n* at risk)	*p*-value	Not upstaged (*n* at risk)	Upstaged (*n* at risk)	*p*-value
Overall survival	12 months	93.4% (*n* = 107)	68.0% (*n* = 18)	< 0.01	93.1% (*n* = 75)	68.0% (*n* = 18)	< 0.01
	36 months	82.5% (*n* = 48)	34.9% (*n* = 10)	< 0.01	79.8% (*n* = 33)	34.9% (*n* = 10)	< 0.01
	60 months	67.5% (*n* = 18)	25.5% (*n* = 3)	< 0.01	58.6% (*n* = 12)	25.5% (*n* = 3)	< 0.01
Disease-specific survival	12 months	93.4% (*n* = 107)	68.0% (*n* = 18)	< 0.01	93.1% (*n* = 75)	68.0% (*n* = 18)	< 0.01
	36 months	83.4% (*n* = 48)	34.9% (*n* = 10)	< 0.01	81.1% (*n* = 33)	34.9% (*n* = 10)	< 0.01
	60 months	73.1% (*n* = 18)	25.5% (*n* = 3)	< 0.01	66.2% (*n* = 12)	25.5% (*n* = 3)	< 0.01
Disease-free survival	12 months	88.7% (*n* = 102)	61.2% (*n* = 14)	< 0.01	86.3% (*n* = 70)	61.2% (*n* = 14)	< 0.01
	36 months	72.8% (*n* = 40)	24.6% (*n* = 5)	< 0.01	67.1% (*n* = 26)	24.6% (*n* = 5)	< 0.01
	60 months	65.7% (*n* = 13)	14.8% (*n* = 2)	< 0.01	56.8% (*n* = 8)	14.8% (*n* = 2)	< 0.01

Statistics presented: *p*-values are calculated with log-rank tests with administrative censoring at predefined time points (12, 36, and 60 months)

**Fig. 2:**
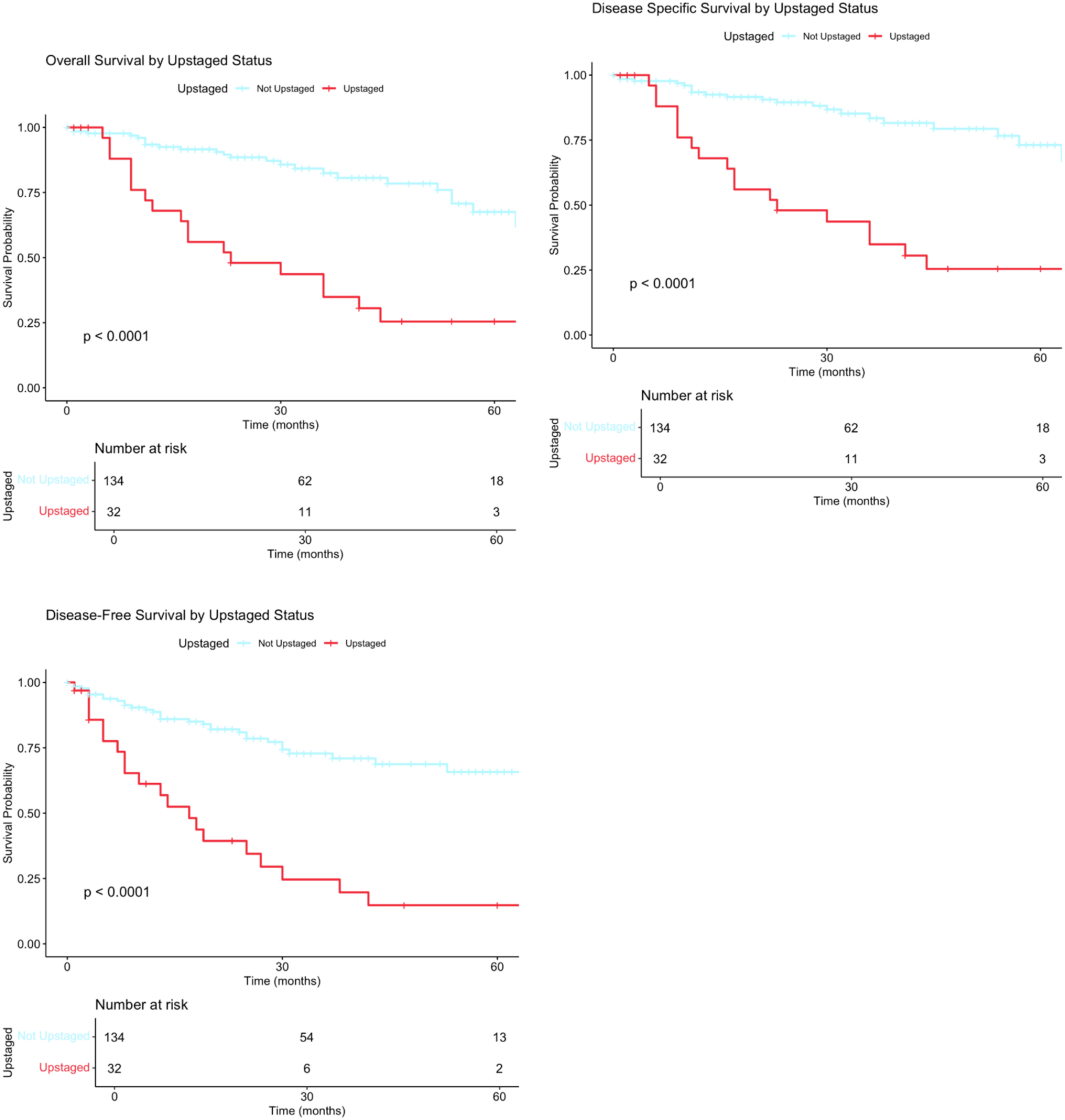
Survival analysis

Survival analysis using Kaplan–Meier method demonstrated a statistically significant prognostic effect of upstaging to advanced FIGO stages III-IV following RALS on long-term survival outcomes in patients with clinically early-stage EC at a high-intermediate-risk or high-risk for recurrence. Patients reclassified as having advanced stage FIGO III or IV disease had a statistically significant worse OS (*p* < 0.01), DSS (*p* < 0.01) and DFS (*p* < 0.01) in contrast to patients who remained classified as early-stage disease (Fig. 2). In multivariable Cox regression adjusting for age, LVSI, and myometrial invasion, upstaging remained independently associated with worse overall survival (HR 2.77, 95% CI 1.40—5.48, *p* = 0.003), disease-specific survival (HR 3.21, 95% CI 1.57—6.54, *p* = 0.001) and disease-free survival (HR 2.06, 95% CI 1.09—3.92, *p* = 0.027) (Supplementary 2).

In total, 52 patients (31.3%) had a recurrence of disease. Of the 32 patients who were upstaged to advanced stage FIGO III-IV, 20 patients (62,5%) had a recurrence of disease. Of the 134 patients who remained classified as FIGO stage I-II, 32 patients (23.9%) had disease recurrence. The median time until recurrence of these patients was 14 months (IQR 7.50–28.00). 17/32 (53.1%) patients had unifocal recurrence of disease. 3/32 patients (9.4%) had port site metastasis, all of whom had additional locations of disease recurrence. Further information on lymph node and organ recurrences in patients who remained classified as FIGO stage I-II are presented in Supplementary 3.

Of the 166 included patients, risk stratification identified 34 patients (20.5%) as intermediate-risk, 32 patients (19.3%) as high-intermediate-risk and 94 patients (56.5%) as high-risk for recurrence (Table 2). This is mainly explained by the absence of preoperative MRI, resulting in 34 patients who were found to have < 50% myometrial invasion only after surgery. In three cases, the postoperative differentiation grade was lower than the preoperative assessment.

All 32 patients who were upstaged postoperatively were classified as high-intermediate risk or high risk (32/126, 25.4%). In this combined postoperative high-(intermediate-)risk groups, OS rates for the 94 patients who remained FIGO stages I-II at 1, 3, and 5-year follow-up were 93.1%, 79.8% and 58.6% and DSS rates were 93.1%, 81.1%, and 66.2%. OS and DSS rates for the 32 patients who were reclassified as having advanced stage FIGO III-IV did not differ from those previously reported for the entire cohort, as these patients represent the same subgroup (Table 3). In total, 47 of 126 patients (37.3%) in the combined postoperative high-(intermediate-)risk groups had a recurrence of disease. Of the 94 patients who remained early-stage FIGO I-II, 20 patients (21.3%) had a recurrence of disease. Among patients with EEC (*n* = 64), 7 patients (10.9%) were reclassified as having advanced stage FIGO III-IV disease following RALS (Supplementary 4). Upstaging was not associated with a significant difference for both OS (*p* = 0.59) and DSS (*p* = 0.61), in contrast with patients who remained early-stage FIGO I-II EEC. In contrast, DFS was significantly lower in the upstaged group (*p* < 0.01), indicating a higher risk of recurrence without an apparent effect on long-term survival (Supplementary 5).

In 90 patients with non-endometrioid carcinoma, 24 patients (26.7%) were reclassified as having FIGO stage III-IV disease following RALS (Supplementary 4). 19.2% of patients with SC (10/52), was reclassified as having advanced stage FIGO III-IV disease (Supplementary 6). Among patients with SC, those reclassified as having advanced stage FIGO III-IV disease had a statistically significantly worse OS (*p* < 0.01), DSS (*p* < 0.01) and DFS (*p* < 0.01) than those who remained early stage FIGO I-II (Supplementary 7).

Four patients diagnosed with CCC were reclassified as having advanced stage FIGO III-IV disease following RALS and had a statistically significantly worse OS (*p* < 0.01) and DSS (*p* < 0.01) in contrast to those remaining early stage FIGO I-II. In contrast, no significant difference in DFS was observed between the groups (*p* = 0.14), though these findings should be interpreted with caution due to the small subgroup size (*n* = 11) (Supplementary 8 & 9).

37% of patients with CS (10/27) were reclassified as having advanced stage FIGO III-IV disease following RALS (Supplementary 10) Upstaging was not associated with a significant difference in OS (*p* = 0.09) or DSS (*p* = 0.06). In contrast, DFS was statistically significant worse in upstaged patients (*p* = 0.05) in contrast to those who remained classified as early-stage disease, although this finding may be influenced by the limited subgroup size (*n* = 27) (Supplementary 11).

Postoperative histopathological assessment identified CS in four patients (2.4%). Other histological subtypes were diagnosed in an additional six patients (3.6%) (Table 2) This group consisted of two patients with rhabdomyosarcoma, one patient with adenosarcoma, one patient with mesonephric-like adenocarcinoma, one patient with mucinous carcinoma, and one patient with neuroendocrine carcinoma.

## Discussion

This study evaluates RALS in 166 high-intermediate and high-risk EC patients resulting in 19.3% upstaging. 5-year DSS for patients who reclassified as having advanced FIGO stages III-IV following RALS was 25.5% compared to 73.1% for patients who were not upstaged. Notably, the majority of not upstaged patients underwent VBT and the majority upstaged patients underwent either EBRT or chemotherapy. During the study period, combined chemoradiotherapy was not routinely administered, as this was not our institutional standard.

OS and DSS did not differ significantly between upstaged and non-upstaged patients with EEC and CS, despite a significant reduction in their DFS. This observation in patients with CS should be interpreted with caution given the limited sample size (*n* = 27). For patients with EEC this may be explained by the fact that the risk groups in this study differed from the current updated risk classification. This subgroup may include POLE-mutated tumours, whose generally favourable clinical course could contribute to the favourable survival outcomes reported in this study [16]. In this cohort, the recurrence rate was 31.3%, similar to the 27.4%–44.8% range reported in the PORTEC-3 study [17]. The most frequent locations of recurrence were the pelvis (11.2%, of which 3/15 patients had vaginal recurrence), peritoneum (11.2%) or lungs (7.5%).

This study demonstrates that the added value of surgical staging without performing molecular classification lies in the prognostic information it provides regarding patient outcomes. Especially for patients who are not upstaged after surgical staging 5-years survival rate is nearly 75%. Upstaging remained independently associated with worse survival outcomes in multivariable Cox regression analyses, underscoring its prognostic significance. The question remains how this prognostic information can be translated to improved adjuvant treatment for upstaged patients and whether there are patients who should receive adjuvant therapy regardless of the results of staging surgery. Hopefully, the molecular classification will improve surgical and adjuvant treatment strategies and provide new therapeutic targets.

RALS is associated with fewer morbidities such as lymphedema and lymphoceles, compared to laparotomic staging [15, 18, 19]. To further reduce the morbidity of staging surgery, SLN procedure is increasingly being adopted by gynaecologic oncologists as a replacement for systematic lymphadenectomy. Various systematic reviews found that no statistically significant differences in survival were observed between patients who underwent SLN and lymphadenectomy [20–25]. However, several of these reviews were assessed as low quality and did not explicitly describe survival outcomes of SLN in patients with EC at a high-intermediate- or high-risk for recurrence [26]. The SENTIREC-ENDO trial demonstrated a safe diagnostic algorithm for SLN mapping in high-risk EC [27]. A limitation however of the SLN procedure is the risk to miss isolated paraaortic metastases [28, 29]. In this study cohort 5/166 patients (3%) had isolated paraaortic metastases. PALND was not performed mainly due to technical feasibility in a subset of patients (30/166, 18.1%), which could have led to missed isolated paraaortic lymph node metastasis. Although paraaortic metastasis could be missed, SLN procedure uses ultrastaging which increases the likelihood of detecting lymph node metastasis [30, 31]. Future research may determine whether lymphadenectomy can be omitted or substituted by SLN procedure guided by preoperative molecular risk classification. Arguably SLN procedure will become the standard of care in the near future [32–34].

A strength of this study is the availability of detailed clinicopathological and surgical data, allowing for a comprehensive evaluation of oncological outcomes in a real-world tertiary care setting. Nonetheless, the retrospective study design may limit the broader applicability of our results. A limitation is the heterogenous study population, resulting in small subgroup sizes that limit the ability to draw definite conclusions from our findings. The lack of a control group without comprehensive staging limits the interpretability of our findings to prognostic stratification following RALS and prevents definitive conclusions about therapeutic benefit. Furthermore, the impact of adjuvant treatments could not be adequately evaluated because no control group was available. We cannot conclude that omitting or adding adjuvant EBRT and/or chemotherapy for patients who remained early stage FIGO I-II following RALS would lead to improved oncological outcomes. Adjuvant therapy strategies after RALS differ in national and international practice, which precludes definitive conclusions regarding the best optimal adjuvant treatment after RALS. In our cohort, the majority of patients who were not upstaged underwent VBT postoperatively. For patients who were upstaged, combined chemotherapy and radiotherapy were not routinely administered, which differs from practice in some centres worldwide. Changes in the adjuvant treatment standards during the inclusion period may also have influenced oncological outcomes and may limit the generalisability of these findings to current treatment strategies. Another limitation concerns the variability in staging approaches, as the SLN procedure has already been implemented in several centres worldwide. Nevertheless, these differences are unlikely to have substantially influenced the observed upstaging rates.

Future research should focus on integrating the clinicopathological factors with molecular risk groups to inform the extent of surgical staging and to optimize adjuvant therapy strategies. The role of molecular risk stratification in informing surgical staging decisions, through evaluation of metastatic patterns across molecular subgroups, is currently being investigated in the prospective EUGENIE trial. A less invasive approach using SLN mapping may be considered for patients with EC at a high-intermediate- or high-risk for recurrence. Evaluating the clinical integration of molecular classification may allow for patient-specific adjuvant therapy decisions, ultimately contributing to improved survival outcomes.

## Conclusions

This cohort study contributes to a better understanding of prognosis in patients with clinically early-stage EC at high-intermediate or high-risk for recurrence, who underwent surgically staging with RALS. We found that 5-year DSS for upstaged patients was 25.5% compared to 73.1% for patients who were not upstaged. RALS provides important prognostic information, while its therapeutic benefit warrants further investigation. Prospective research should assess the impact of adjuvant treatment strategies guided by surgical staging. Identifying patient subgroups using molecular risk classification, including patients with POLEmut EEC and a favourable prognosis, for whom RALS or adjuvant therapy may offer none or little benefit, or patients with aggressive p53abn EC who should possibly receive adjuvant treatment regardless of surgical staging outcomes, could help redefine treatment protocols.

## Supplementary Information

Below is the link to the electronic supplementary material.

Supplementary file1

## Data Availability

Metadata describing the participant data analyzed in this study are available through the HDSU catalogue (https://catalogue.hdsu.nl/HDSU/collections/RobEcO). Participant data was extracted from electronic medical patient files. Due to the sensitive and protected nature of the underlying data, the raw datasets are not publicly available. Access to the data may be granted upon reasonable request, subject to approval of a research proposal and completion of a data-sharing agreement.
