# Supplementary material for: Long-term prognostic value of staging surgery for high-intermediate-risk and high-risk endometrial cancer
Source: J Robot Surg. 2026 Apr 27;20(1):468. doi: 10.1007/s11701-026-03316-6 (PMC13121418; doi:10.1007/s11701-026-03316-6)

SUPPLEMENTARY MATERIAL

**Supplement 1:** Postoperative pathology characteristics of patients upstaged to FIGO stage III-IV and patients not upstaged

| **Characteristic** | **Patients upstaged to FIGO stage III-IV, *n* = 32** | **Patients not upstaged, *n* = 134** |
| --- | --- | --- |
| Age at intake (years) | 69 (65, 75) | 68 (60, 73) |
| Postoperative histology |  |  |
| *Endometrioid carcinoma* | 7 (21.9%) | 57 (42.5%) |
| *Serous carcinoma* | 10 (31.3%) | 42 (31.3%) |
| *Clear cell carcinoma* | 4 (12.5%) | 7 (5.2%) |
| *Carcinosarcoma* | 10 (31.3%) | 17 (12.7%) |
| *Undifferentiated carcinoma* | 1 (3.1%) | 3 (2.2%) |
| *Tumour free* | 0 (0%) | 2 (1.5%) |
| Lymph node metastasis |  |  |
| *Yes* | 21 (65.6%) | 0 (0%) |
| *Micrometastasis* | 18 (56.3%) | 0 (0%) |
| *Macrometastasis* | 3 (9.4%) | 0 (0%) |
| *No* | 9 (28.1%) | 132 (98.5%) |
| *Not performed* | 2 (6.3%) | 2 (1.5%) |
| Omental metastasis |  |  |
| *Yes* | 5 (15.6%) | 0 (0%) |
| *No* | 20 (62.5%) | 83 (61.9%) |
| *Not performed* | 7 (21.9%) | 51 (38.1%) |
| Peritoneal metastasis |  |  |
| *Yes* | 5 (15.6%) | 0 (0%) |
| *No* | 15 (46.9%) | 71 (53.0%) |
| *Not performed* | 12 (37.5%) | 63 (47.0%) |
| LVSI | 20 (62.5%) | 31 (23.3%) |
| *Unknown* |  | 1 |
| Adjuvant therapy |  |  |
| *None* | 6 (18.8%) | 19 (14.2%) |
| *Vaginal brachytherapy* | 1 (3.1%) | 94 (70.1%) |
| *External beam radiotherapy* | 13 (40.6%) | 17 (12.7%) |
| *Chemotherapy* | 9 (28.1%) | 2 (1.5%) |
| *Other* | 3 (9.4%) | 2 (1.5%) |
| Statistics presented: median (IQR); n (%) | |  |
| Abbreviations: FIGO = International Federation of Gynecology and Obstetrics; LVSI = lymphovascular space invasion, IQR = interquartile range. | |  |

Supplement 2a: Multivariable Cox proportional hazards analysis for overall survival

| **Characteristic** | **HR** | **95% CI** | **p-value** | |
| --- | --- | --- | --- | --- |
| Upstaging |  |  |  | |
| No | — | — |  | |
| Yes | 2.77 | 1.40, 5.48 | 0.003 | |
| Age at intake (years) | 1.05 | 1.00, 1.10 | 0.032 | |
| Lymphovascular space invasion (LVSI) |  |  |  | |
| No | — | — |  | |
| Yes | 2.32 | 1.21, 4.44 | 0.011 | |
| Myometrial invasion |  |  |  | |
| <50% | — | — |  | |
| >50% | 0.89 | 0.44, 1.77 | 0.7 | |
| Abbreviations: CI = Confidence Interval, HR = Hazard Ratio | | | |  |

Supplement 2b: Univariable Cox proportional hazards analysis for disease-specific survival

| **Characteristic** | **HR** | **95% CI** | **p-value** | |
| --- | --- | --- | --- | --- |
| Upstaging |  |  |  | |
| No | — | — |  | |
| Yes | 3.21 | 1.57, 6.54 | 0.001 | |
| Age at intake (years) | 1.06 | 1.01, 1.11 | 0.026 | |
| Lymphovascular space invasion (LVSI) |  |  |  | |
| No | — | — |  | |
| Yes | 2.03 | 1.02, 4.03 | 0.043 | |
| Myometrial invasion |  |  |  | |
| <50% | — | — |  | |
| >50% | 0.91 | 0.44, 1.87 | 0.8 | |
| Abbreviations: CI = Confidence Interval, HR = Hazard Ratio | | | |  |

| **Characteristic** | **HR** | **95% CI** | **p-value** |
| --- | --- | --- | --- |
| Upstaging |  |  |  |
| No | — | — |  |
| Yes | 2.06 | 1.09, 3.92 | 0.027 |
| Age at intake (years) | 1.05 | 1.01, 1.09 | 0.026 |
| Lymphovascular space invasion (LVSI) |  |  |  |
| No | — | — |  |
| Yes | 3.12 | 1.71, 5.71 | <0.001 |
| Myometrial invasion |  |  |  |
| <50% | — | — |  |
| >50% | 0.98 | 0.51, 1.87 | >0.9 |
| Abbreviations: CI = Confidence Interval, HR = Hazard Ratio | | | |

Supplement 2c: Multivariable Cox proportional hazards analysis for disease-free survival

Supplement 3: Recurrence patterns of patients who remained FIGO stage I-II


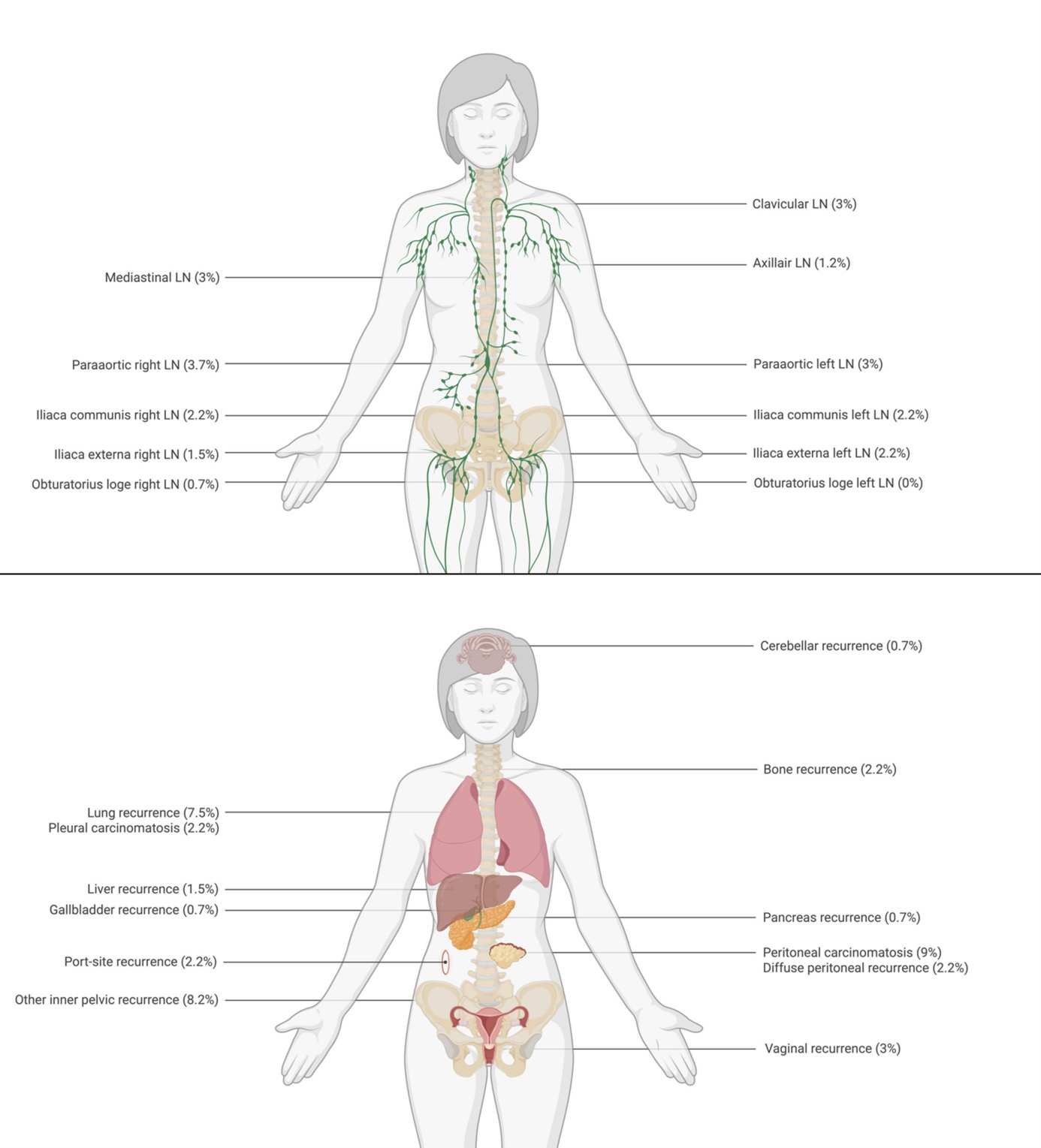


**Supplement 4:** Characteristics of patients with endometrioid and non-endometrioid carcinoma

| **Characteristic** | **Patients with endometrioid carcinoma, *n* = 64** | **Patients with non-endometrioid carcinoma^1^, *n* = 90** |
| --- | --- | --- |
| Preoperative FIGO stage |  |  |
| *IA* | 56 (87.5%) | 75 (83.3%) |
| *IB* | 4 (6.3%) | 8 (8.9%) |
| *II* | 4 (6.3%) | 7 (7.8%) |
| Postoperative FIGO stage |  |  |
| *IA* | 34 (53.1%) | 36 (40.0%) |
| *IB* | 18 (28.1%) | 14 (15.6%) |
| *II* | 5 (7.8%) | 16 (17.8%) |
| *IIIA* | 2 (3.1%) | 1 (1.1%) |
| *IIIB* | 1 (1.6%) | 2 (2.2%) |
| *IIIC1* | 3 (4.7%) | 7 (7.8%) |
| *IIIC2* | 1 (1.6%) | 6 (6.7%) |
| *IVA* | 0 (0%) | 2 (2.2%) |
| *IVB* | 0 (0%) | 6 (6.7%) |
| Lymph node metastasis |  |  |
| *Yes* | 4 (6.3%) | 16 (17.8%) |
| *Micrometastasis* | 4 (6.3%) | 13 (8.7%) |
| *Macrometastasis* | 0 (0%) | 3 (1.8%) |
| *No* | 59 (92.2%) | 71 (78.9%) |
| *Not performed* | 1 (1.6%) | 3 (3.3%) |
| Omental metastasis |  |  |
| *Yes* | 0 (0%) | 5 (5.6%) |
| *No* | 14 (21.9%) | 79 (87.8%) |
| *Not performed* | 50 (78.1%) | 6 (6.7%) |
| Peritoneal metastasis |  |  |
| *Yes* | 0 (0%) | 5 (5.6%) |
| *No* | 21 (32.8%) | 57 (63.3%) |
| *Not performed* | 43 (67.2%) | 28 (31.1%) |
| Adjuvant therapy |  |  |
| *None* | 9 (14.1%) | 10 (11.1%) |
| *Vaginal brachytherapy* | 46 (71.9%) | 45 (50.0%) |
| *External beam radiotherapy* | 8 (12.5%) | 21 (23.3%) |
| *Chemotherapy* | 0 (0%) | 10 (11.1%) |
| *Other* | 1 (1.6%) | 4 (4.4%) |
| Statistics presented: median (IQR); n (%)  ^1^ Serous carcinoma, clear cell carcinoma and carcinosarcoma  Abbreviations: FIGO = International Federation of Gynecology and Obstetrics, IQR = interquartile range. | |  |


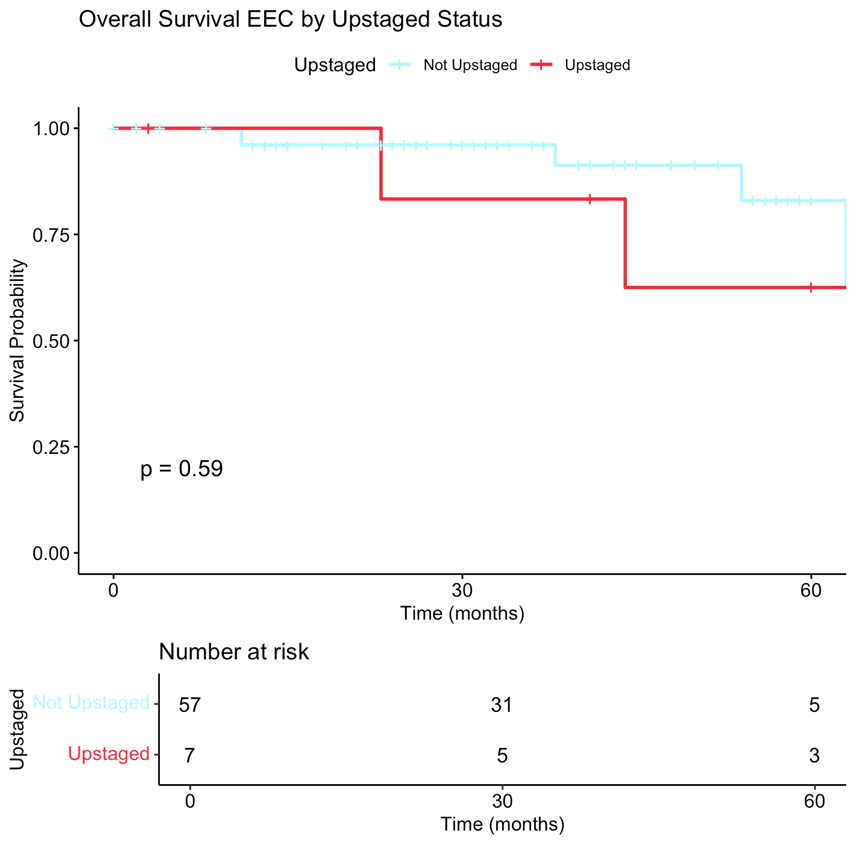
Supplement 5: Survival analysis endometrioid carcinoma


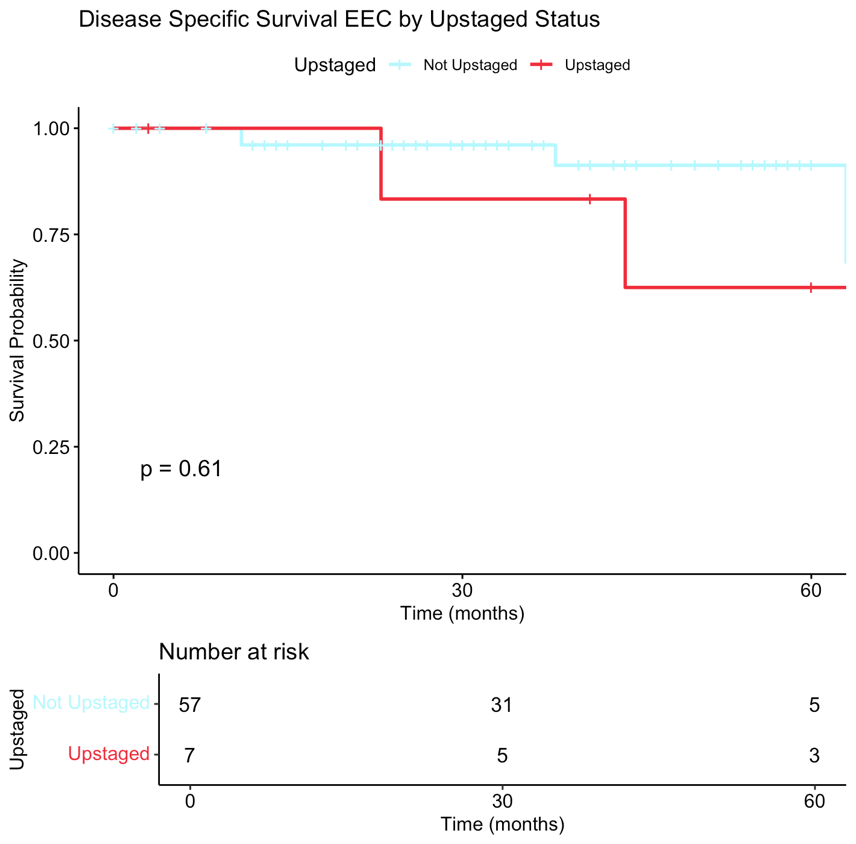

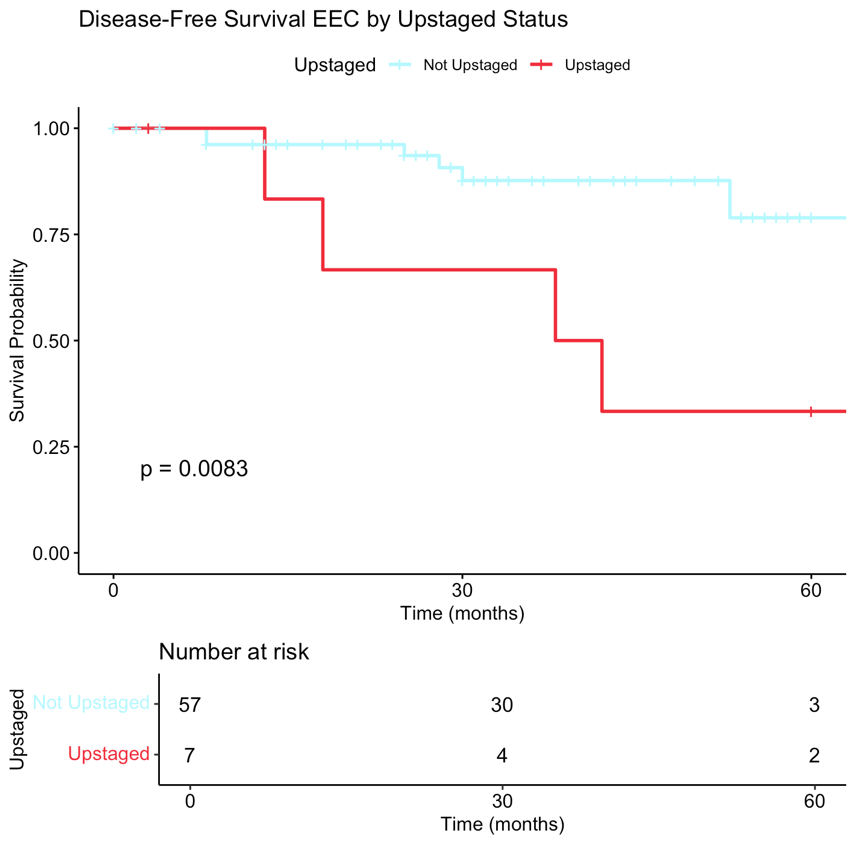


**Supplement 6:** Characteristics of patients with serous carcinoma

| **Characteristic** | **Study population, *n* = 52** |
| --- | --- |
| Preoperative FIGO stage |  |
| *IA* | 46 (88.5%) |
| *IB* | 3 (5.8%) |
| *II* | 3 (5.8%) |
| Postoperative FIGO stage |  |
| *IA* | 22 (42.3%) |
| *IB* | 9 (17.3%) |
| *II* | 11 (21.2%) |
| *IIIC1* | 3 (5.8%) |
| *IIIC2* | 1 (1.9%) |
| *IVA* | 1 (1.9%) |
| *IVB* | 5 (9.6%) |
| Lymph node metastasis |  |
| *Yes* | 6 (11.5%) |
| *Micrometastasis* | 5 (9.6%) |
| *Macrometastasis* | 1 (1.9%) |
| *No* | 43 (82.7%) |
| *Not performed* | 3 (5.8%) |
| Omental metastasis |  |
| *Yes* | 4 (7.7%) |
| *No* | 47 (90.4%) |
| *Not performed* | 1 (1.9%) |
| Peritoneal metastasis |  |
| *Yes* | 4 (7.7%) |
| *No* | 38 (73.1%) |
| *Not performed* | 10 (19.2%) |
| Adjuvant therapy |  |
| *None* | 5 (9.6%) |
| *Vaginal brachytherapy* | 30 (57.7%) |
| *External beam radiotherapy* | 8 (15.4%) |
| *Chemotherapy* | 8 (15.4%) |
| *Other* | 1 (1.9%) |
| Statistics presented: median (IQR); n (%) | |
| Abbreviations: FIGO = International Federation of Gynecology and Obstetrics; IQR = interquartile range. | |


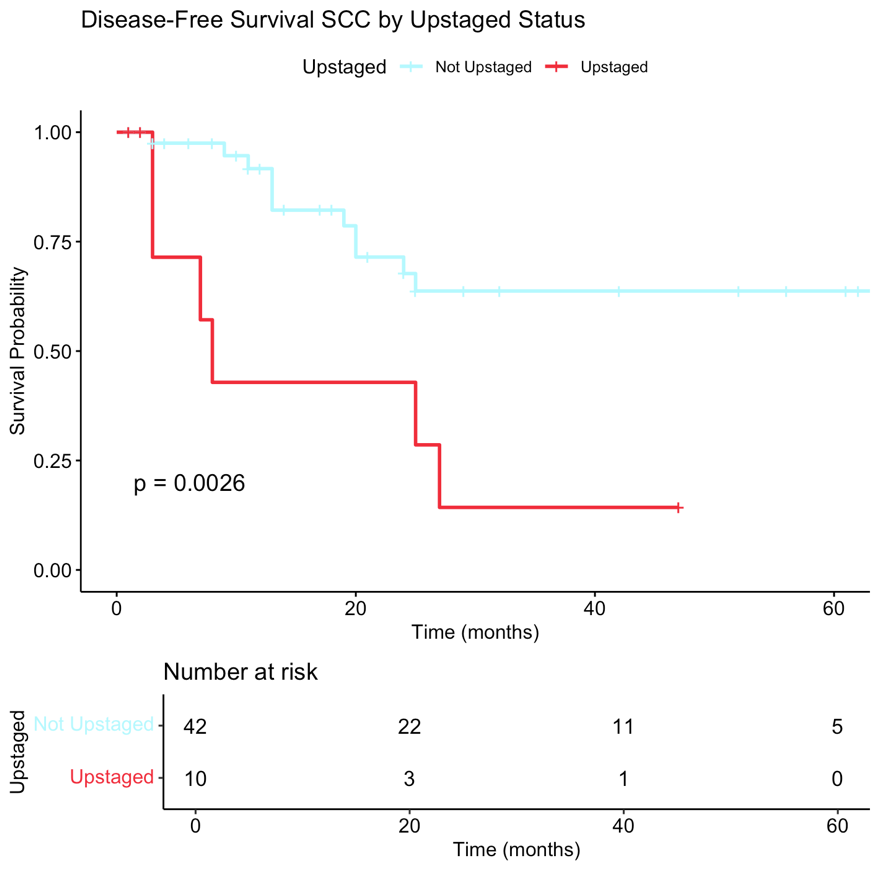

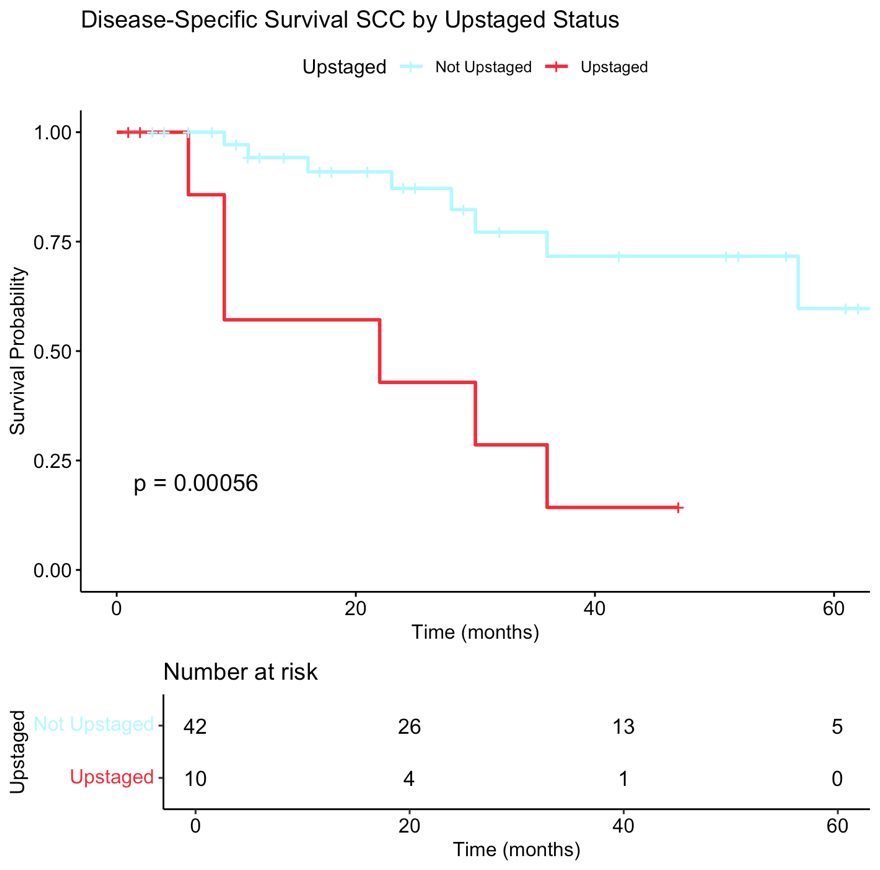

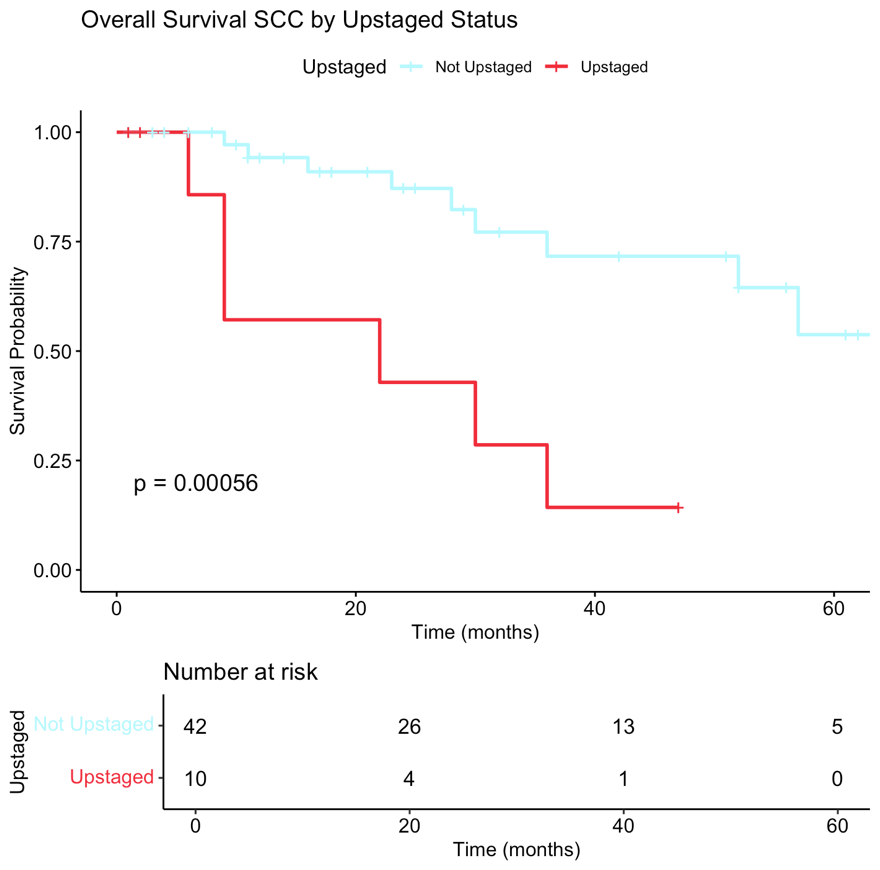
Supplement 7: Survival analysis serous carcinoma

**Supplement 8:** Characteristics of patients with clear cell carcinoma

| **Characteristic** | **Study population, *n* = 11** |
| --- | --- |
| Preoperative FIGO stage |  |
| *IA* | 8 (72.7%) |
| *IB* | 1 (9.1%) |
| *II* | 2 (18.2%) |
| Postoperative FIGO stage |  |
| *IA* | 5 (45.5%) |
| *II* | 2 (18.2%) |
| *IIIB* | 1 (9.1%) |
| *IIIC1* | 1 (9.1%) |
| *IIIC2* | 1 (9.1%) |
| *IVA* | 1 (9.1%) |
| Lymph node metastasis |  |
| *Yes* | 2 (18.2%) |
| *Micrometastasis* | 1 (9.1%) |
| *Macrometastasis* | 1 (9.1%) |
| *No* | 0 (0%) |
| *Not performed* | 9 (81.8%) |
| Omental metastasis |  |
| *Yes* | 0 (0%) |
| *No* | 9 (81.8%) |
| *Not performed* | 2 (18.2%) |
| Peritoneal metastasis |  |
| *Yes* | 1 (9.1%) |
| *No* | 6 (54.5%) |
| *Not performed* | 4 (36.4%) |
| Adjuvant therapy |  |
| *None* | 1 (9.1%) |
| *Vaginal brachytherapy* | 6 (54.5%) |
| *External beam radiotherapy* | 2 (18.2%) |
| *Other* | 2 (18.2%) |
| Statistics presented: median (IQR); n (%) | |
| Abbreviations: FIGO = International Federation of Gynecology and Obstetrics; IQR = interquartile range. | |


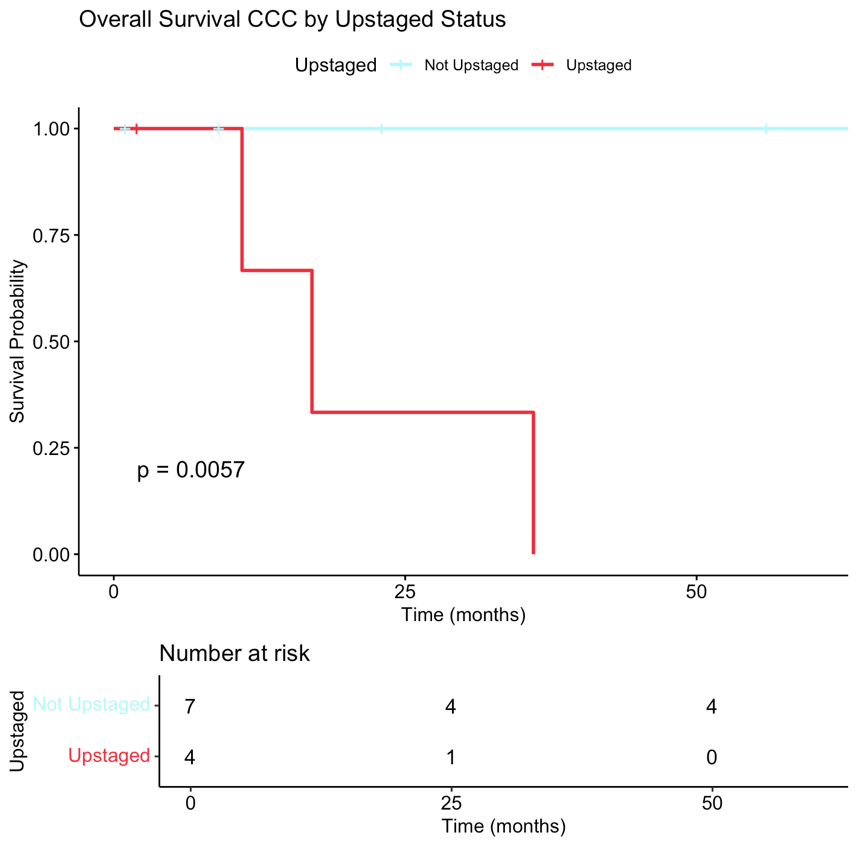

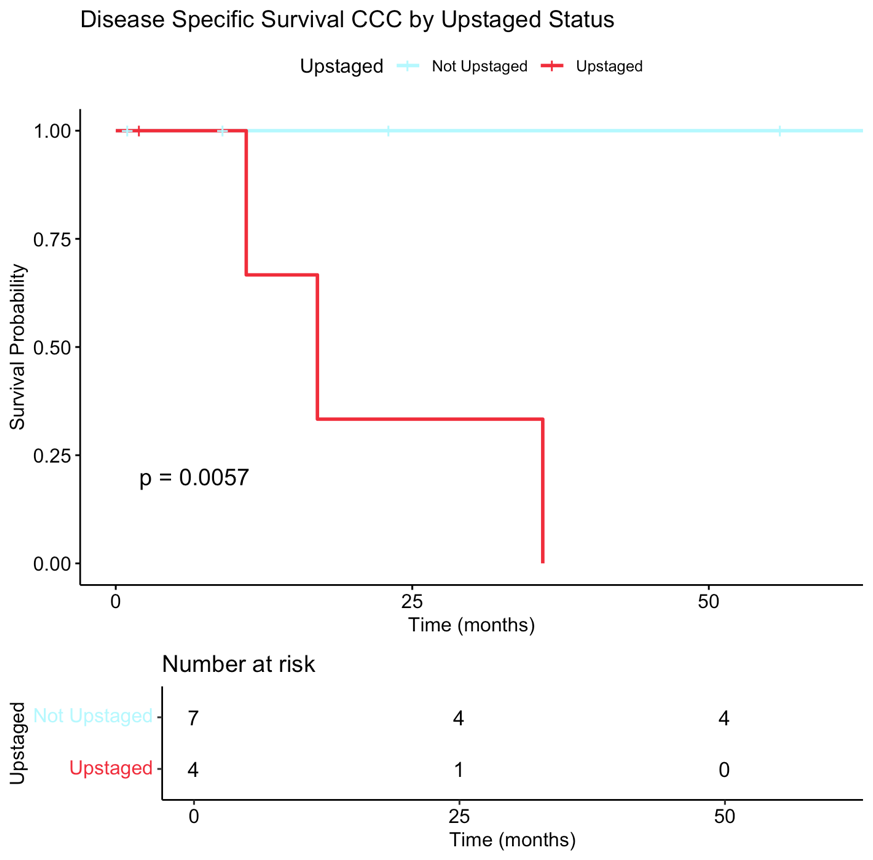
Supplement 9: Survival analysis clear cell carcinoma


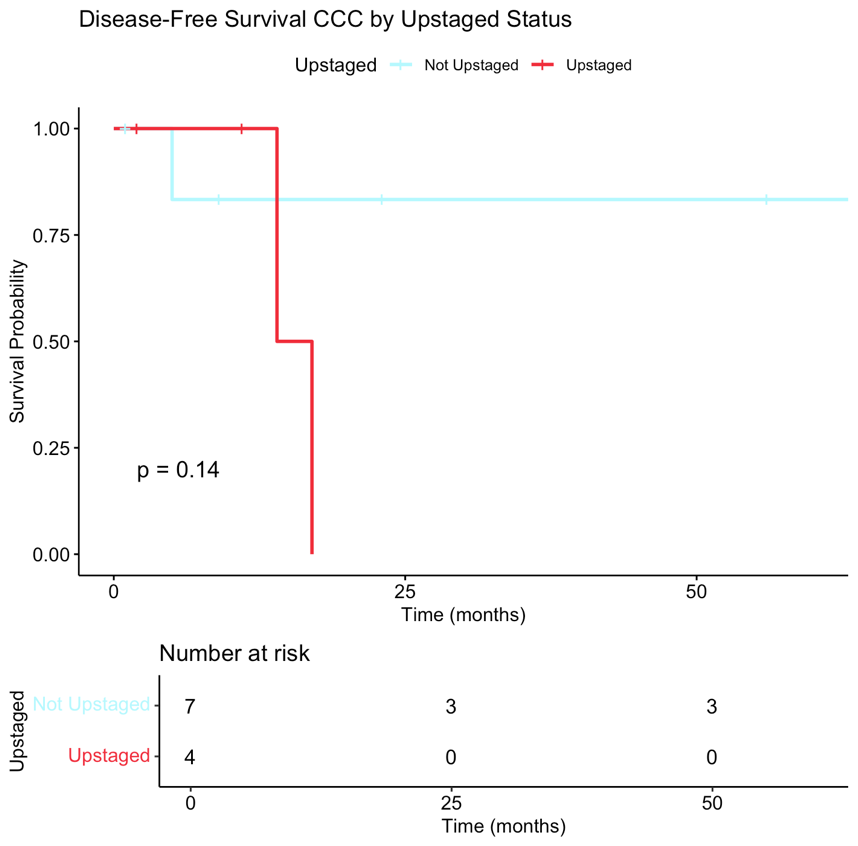


**Supplement 10:** Characteristics of patients with carcinosarcoma

| **Characteristic** | **Study population, *n* = 27** |
| --- | --- |
| Preoperative FIGO stage |  |
| *IA* | 21 (77.8%) |
| *IB* | 4 (14.8%) |
| *II* | 2 (7.4%) |
| Postoperative FIGO stage |  |
| *IA* | 9 (33.3%) |
| *IB* | 5 (18.5%) |
| *II* | 3 (11.1%) |
| *IIIA* | 1 (3.7%) |
| *IIIB* | 1 (3.7%) |
| *IIIC1* | 3 (11.1%) |
| *IIIC2* | 4 (14.8%) |
| *IVB* | 1 (3.7%) |
| Lymph node metastasis |  |
| *Yes* | 8 (29.6%) |
| *Micrometastasis* | 7 (25.9%) |
| *Macrometastasis* | 1 (3.7%) |
| *No* | 0 (0%) |
| *Not performed* | 19 (70.4%) |
| Omental metastasis |  |
| *Yes* | 1 (3.7%) |
| *No* | 23 (85.2%) |
| *Not performed* | 3 (11.1%) |
| Peritoneal metastasis |  |
| *No* | 13 (48.1%) |
| *Not performed* | 14 (51.9%) |
| Adjuvant therapy |  |
| *None* | 4 (14.8%) |
| *Vaginal brachytherapy* | 9 (33.3%) |
| *External beam radiotherapy* | 11 (40.7%) |
| *Chemotherapy* | 2 (7.4%) |
| *Other* | 1 (3.7%) |
| Statistics presented: median (IQR); n (%) | |
| Abbreviations: FIGO = International Federation of Gynecology and Obstetrics; IQR = interquartile range. | |


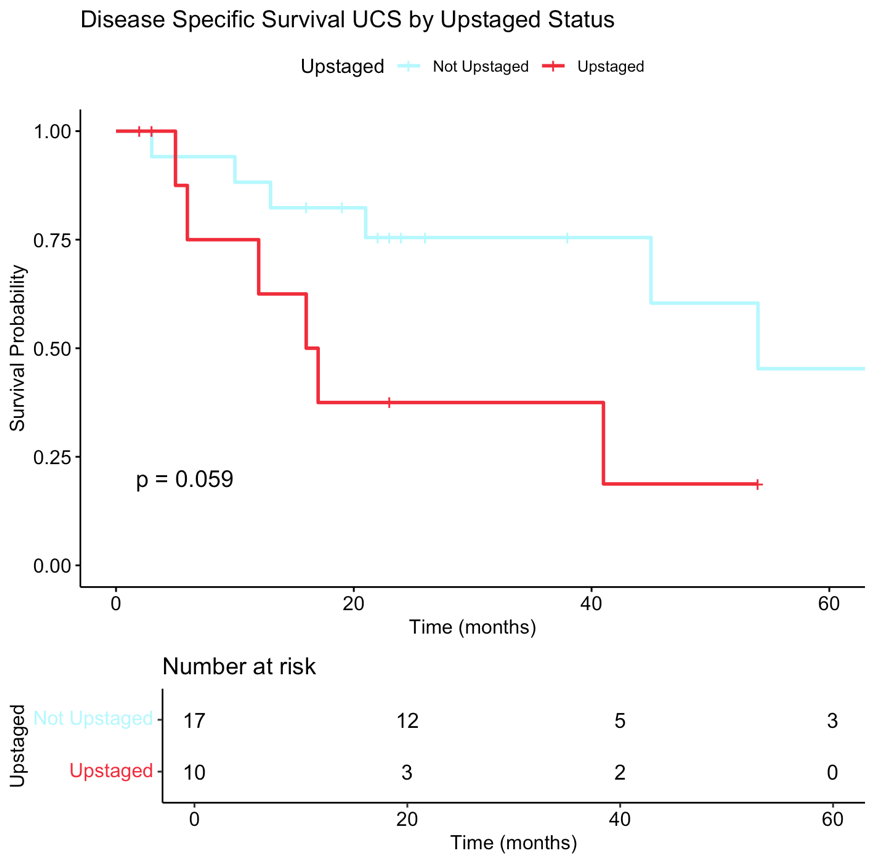

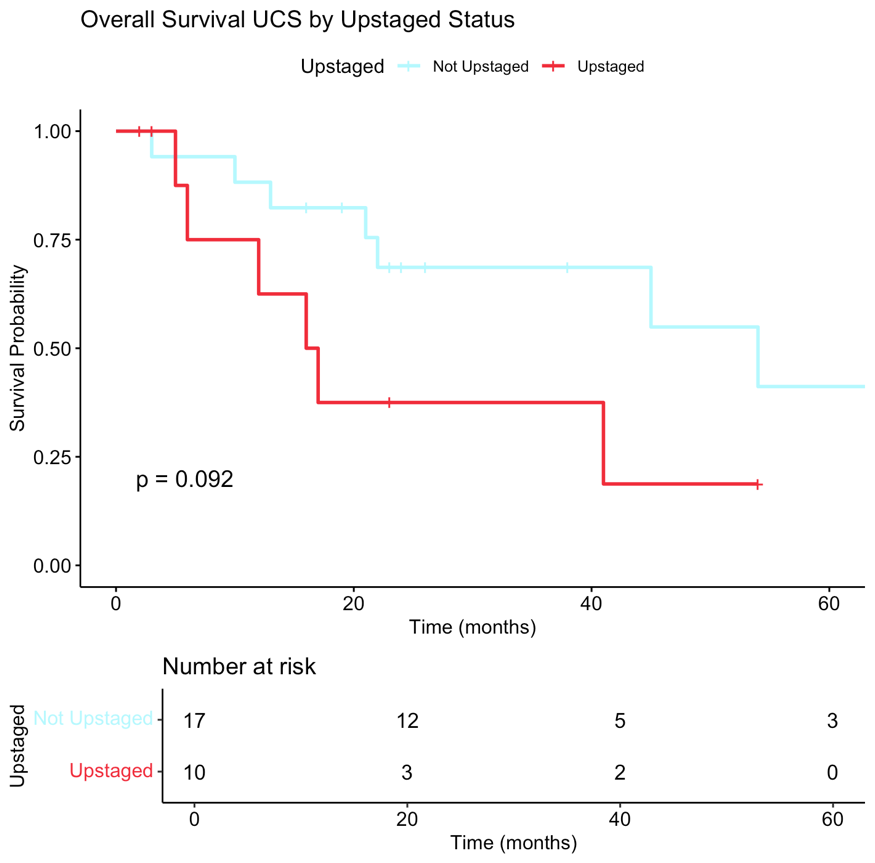
Supplement 11: Survival analysis carcinosarcoma


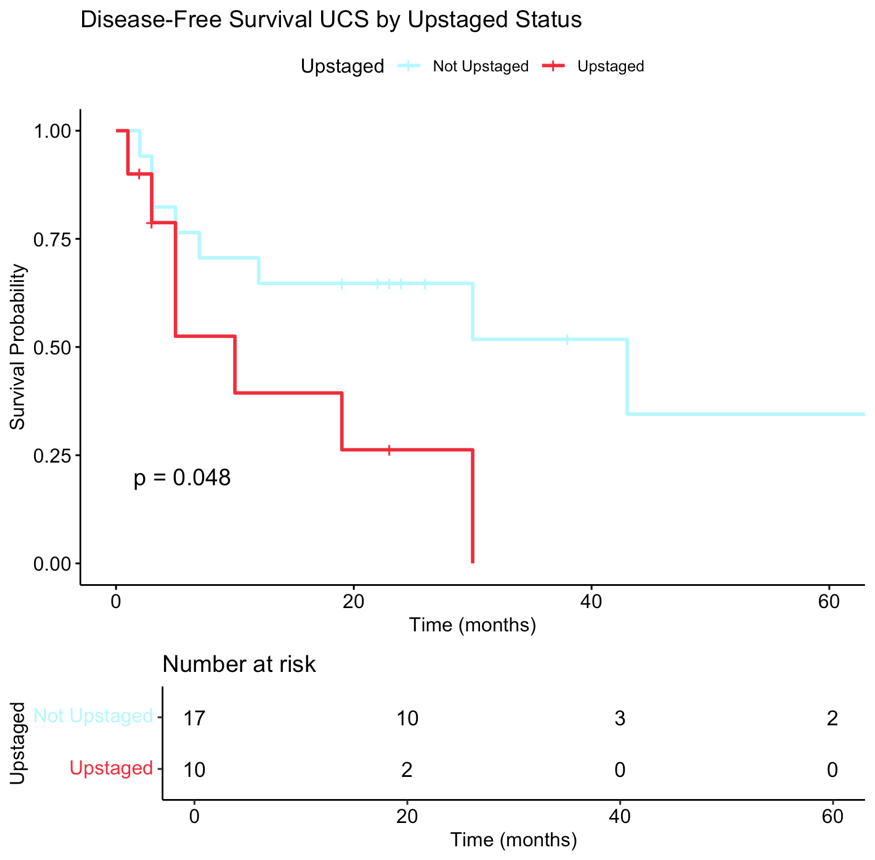

Supplement: Supplementary file 1 — Supplementary file1 [file 11701_2026_3316_MOESM1_ESM.docx]
